# H3K9 methyltransferase G9a negatively regulates UHRF1 transcription during leukemia cell differentiation

**DOI:** 10.1093/nar/gkv183

**Published:** 2015-03-12

**Authors:** Kee-Beom Kim, Hye-Ju Son, Sulji Choi, Ja Young Hahm, Hyeonsoo Jung, Hee Jo Baek, Hoon Kook, Yoonsoo Hahn, Hyun Kook, Sang-Beom Seo

**Affiliations:** 1 Department of Life Science, College of Natural Sciences, Chung-Ang University, Seoul 156-756; 2 Environmental Health Center for Childhood Leukemia and Cancer, Department of Pediatrics, Chonnam National University Hwasun Hospital, Hwasun 519-809; 3 Medical Research Center for Gene Regulation and Department of Pharmacology, Chonnam National University, Gwangju 501-746, Republic of Korea

## Abstract

Histone H3K9 methyltransferase (HMTase) G9a-mediated transcriptional repression is a major epigenetic silencing mechanism. UHRF1 (ubiquitin-like with PHD and ring finger domains 1) binds to hemimethylated DNA and plays an essential role in the maintenance of DNA methylation. Here, we provide evidence that UHRF1 is transcriptionally downregulated by H3K9 HMTase G9a. We found that increased expression of G9a along with transcription factor YY1 specifically represses UHRF1 transcription during TPA-mediated leukemia cell differentiation. Using ChIP analysis, we found that *UHRF1* was among the transcriptionally silenced genes during leukemia cell differentiation. Using a DNA methylation profiling array, we discovered that the *UHRF1* promoter was hypomethylated in samples from leukemia patients, further supporting its overexpression and oncogenic activity. Finally, we showed that G9a regulates UHRF1-mediated H3K23 ubiquitination and proper DNA replication maintenance. Therefore, we propose that H3K9 HMTase G9a is a specific epigenetic regulator of UHRF1.

## INTRODUCTION

The structure of chromatin is dynamically regulated by various posttranslational modifications of the core histones. Those modifications influence the folding and functional status of chromatin and thereby eventually control gene expression (1,2). Among the various modifications, lysine methylation regulates diverse substrates, including histones and non-histone proteins, and correlates with distinct biological outcomes, including transcriptional regulation.

G9a and GLP (G9a-like protein) are homologous histone methyltransferases (HMTases) that mediate methylation of histone H3K9-me1, H3K9-me2 and H3K27 (3). In particular, H3K9 methylation by G9a is an integral component of transcriptional repression for many genes. For example, G9a is essential for early mouse embryo development and embryonic stem cell differentiation (4). In G9a knockout mice, H3K9 methylation is drastically reduced, resulting in severe growth retardation and early lethality, which indicates that G9a plays a crucial role in early mammalian development (4,5). G9a/GLP-dependent DNA methylation in G9a or GLP knockout mice has been reported, although catalytically inactive G9a partially restores the aberrant DNA methylation pattern in G9a^−/−^ cells (6–8). G9a/GLP also methylate non-histone proteins, including p53, CDYL1 and Reptin, and have been shown to automethylate as well (9–12). Interestingly, one report suggested that G9a/GLP activity promotes H3K9-me2 patterning in human hematopoietic stem and progenitor cells (HSPCs) and that its inhibition delays HSPC lineage commitment (13). Furthermore, loss of G9a significantly delayed disease progression and reduced leukemia stem cell frequency in an acute myeloid leukemia mouse model (14).

The E3 ubiquitin ligase UHRF1 is a master regulator of epigenetic modifications due to its ability to recognize modifications of both DNA and histones (15–18). By recognizing hemimethylated DNA, UHRF1 maintains genomic DNA methylation by recruiting DNMT1 to DNA replication sites (15–17,19). Consequently, UHRF1 repression results in global DNA hypomethylation (19–22). UHRF1 epigenetically regulates gene expression with histone deacetylase by binding to methylated histones (23–25). UHRF1 is frequently overexpressed in various human tumors and has an important role in cancer pathogenesis and progression (26–29). In cancer cells, UHRF1 represses several tumor-suppressor genes including *p16INK4A, hMLH1, p21* and *RB* (24). Moreover, UHRF1 promotes ubiquitination-mediated degradation of histones and the tumor-suppressor protein PML (30,31). Another study suggested that UHRF1-dependent histone H3 ubiquitination has a prerequisite role in the maintenance of DNA methylation, indicating an important role for epigenetic regulation in diverse UHRF1 activity (31). Despite its key role in the maintenance of CpG DNA methylation and DNA replication, its transcriptional regulation remains poorly understood.

In the current study, we demonstrate that histone H3K9 methyltransferase G9a negatively regulates the transcription of UHRF1. We further focus on G9a and characterize its transcriptional regulatory role during leukemia cell differentiation. Microarray data identified a subset of G9a target genes, including *UHRF1*. G9a was recruited to the *UHRF1* promoter along with YY1 and was found to function as a corepressor of the target gene. During leukemia cell differentiation, G9a expression increased and UHRF1 expression decreased. ChIP and real-time PCR analysis found that RNA polymerase II (Pol II) and histone acetylation in the *UHRF1* promoter were significantly reduced during 12-*O*-tetradecanoylphorbol-13-acetate (TPA)-mediated leukemia cell differentiation, indicating that the oncogene *UHRF1* was tightly repressed. In addition, G9a was shown to function as an upstream regulator of UHRF1-mediated H3K23 ubiquitination and DNA replication maintenance via epigenetic regulation of UHRF1.

## MATERIALS AND METHODS

### Plasmid constructs

For the luciferase assay, genomic DNA was prepared and the *UHRF1* promoter region (−1921 to +145) was inserted into the pGL3-basic vector (Promega). The *UHRF1* promoter sequence was amplified using the following polymerase chain reaction (PCR) primers: the KpnI site-linked primer, 5′-CGGGGTACCCGGGAAAAGACAGCAAACAAG-3′, as a forward primer and the XhoI site-linked primer, 5′-CCGCTCGAGCCCTGTAGAACAGCCTCTGC-3′, as a reverse primer. pcDNA3.0-Flag-hG9a, pEGFP-hG9a, pEGFP-hG9a-ΔSET, pCMV-Flag-YY1, pCMX-HDAC1, and pCMX-HDAC2 were used in each experiment. Short hairpin RNAs (shRNAs) against G9a (RHS4533-NM-006709) were purchased from Open Biosystems. Small interfering RNAs (siRNAs) against YY1 (sc-36863 and sc-44330) were purchased from Santa Cruz Biotechnology.

### Cell culture and synchronization

K562, H1299, and HL-60 cells were grown in RPMI 1640, and G9a^−/−^ MEF and 293T cells were cultured in Dulbecco's modified Eagle's medium supplemented with 10% Fetal bovine serum (FBS). G1/S synchronization was achieved by a double thymidine block. In brief, cells were cultured in the presence of 2 mM thymidine for 19 h and then released to grow for 10 h. Cells were then treated for another 15 h with 2 mM thymidine, causing the cells to arrest at the G1/S boundary. The arrested cells were allowed to enter the S phase by washing the thymidine away with phosphate buffered saline (PBS).

### RNA interference

DNA oligonucleotides encoding G9a shRNA (5′-CACACATTCCTGACCAGAGAT-3′) were subcloned into pLKO.1-puro (Addgene) lentiviral vector according to standard procedures. To produce virus particles, 293T cells were cotransfected with plasmids encoding VSV-G, NL-BH and the shRNAs. Two days after transfection, the soups containing the viruses were collected and used to infect cancer cells in the presence of polybrene (8 μg/ml).

### Generation of stable cell lines

For the expression of doxycycline inducible shG9a RNA, shRNA oligonucleotides against G9a were cloned into the pSingle-tTS-shRNA vector. Independent shG9a RNA oligonucleotides were designed with a 5′-XhoI restriction site overhang on the top strand and a 5′-HindIII restriction site overhang on the bottom strand. Each strand contained hairpin loop (TTCAAGAGA), terminator (TTTTTT), and test restriction sites (MluI, ACGCGT). Doxycycline inducible shG9a RNA was used to generate stable shG9a RNA-expressed K562 cells and microarrays. ShG9a RNA oligonucleotide sequences were as follows: for shG9a #1, 5′-TCGAGAGCTGAATGGGATGGTCTTTTCAAGAGAAAGACCATCCCATTCAGCTTTTTTTACGCGTA-3′ (top strand) and 5′-AGCTTACGCGTAAAAAAAGCTGAATGGGATGGTCTTTCTCTTGAAAAGACCATCCCATTCAGCTC-3′ (bottom strand); for shG9a #2, 5′-TCGAGGTCCAGGAATTTAACAAGATTTCAAGAGAATCTTGTTAAATTCCTGGATTTTTTACGCGTA-3′ (top strand) and 5′-AGCTTACGCGTAAAAAATCCAGGAATTTAACAAGATTCTCTTGAAATCTTGTTAAATTCCTGGACC-3′ (bottom strand). K562 (5 × 10^6^) cells were seeded into a 60-mm dish and transfected with pSingle-tTs-shRNA-G9a using Lipofectamine 2000 (Invitrogen), after which the stably transfected cells were selected in media containing 2.5 mg/ml of G418 (Sigma). Cells were cultured in RPMI in the presence or absence of doxycycline (Sigma) supplemented with 10% of Tet-system-proved FCS (Clontech), which is a tetracycline-free serum developed for tetracycline-controllable expression systems.

### Transcriptional activity assay

For the transcriptional activity assay, 293T cells were seeded in 48-well plates and cotransfected with the indicated expression plasmid and the pGL3-*UHRF1* promoter (−1921 to +145) reporter plasmid using Lipofectamine 2000 (Invitrogen). After 48 h, the cells were harvested and subjected to a luciferase assay (Promega). The level of β-galactosidase activity was used to normalize the reporter luciferase. Data are expressed as the means of four replicates from a single assay. All results shown are representative of at least three independent experiments.

### Histone acid extraction

Cell pellets were resuspended in PBS with 0.5% Triton X-100 and protease inhibitors, and the tubes were subsequently incubated at 4°C for 30 min to lyse the cells. The lysates were centrifuged at 4°C for 10 min at 10,000g, and the pellets were resuspended in 0.2 N HCl. The samples were then centrifuged at 4°C for 10 min at 16,000g. The pellets were again resuspended in 100% TCA and centrifuged at 4°C for 10 min at 16,000g. The histone-containing pellets were collected and eluted in distilled water.

### Immunoprecipitation of chromatin-bound histone H3

Extracted histones were immunoprecipitated with anti-H3 antibody in IP buffer [50 mM Tris–HCl (pH 7.5), 150 mM NaCl, 1 mM ethylenediaminetetraacetic acid (EDTA), 1 mM ethylene glycol tetraacetic acid (EGTA), 1% Triton X-100, 1 mM phenylmethylsulfonyl fluoride (PMSF), and 1× protease inhibitor cocktail] overnight at 4°C. Protein A/G agarose beads (GenDEPOT) were then added for 2 h with agitation at 4°C. Bound proteins were eluted and analyzed by immunoblotting with anti-H3 and anti-Ub antibodies.

### ChIP and real-time PCR analysis

Cells were harvested and subsequently cross-linked with 1% formaldehyde. Briefly, 1% formaldehyde was added to the medium for 10 min at room temperature, followed by the addition of 125 mM glycine for 5 min at room temperature. Adherent cells were scraped from the dishes into 1 ml PBS. The scraped cells were centrifuged, and the resulting pellets were washed once with 1× PBS. The cell pellets were resuspended in sodium dodecyl sulphate (SDS) lysis buffer [1% SDS, 10 mM EDTA, 50 mM Tris–HCl (pH 8.1)]. Cells were then sonicated, and the lysates were subjected to immunoprecipitation using the indicated antibodies. The immunoprecipitates were eluted and reverse cross-linked, after which the DNA fragments were purified for polymerase chain reaction (PCR) amplification. To analyze the *UHRF1* promoter region, primer sets consisting of the 153 bp region (nucleotides −166 to −14; sense, 5′-GGCTGTACAGGAGGACTGGA-3′, and antisense, 5′-AGCAAAAACCCCCATCAGTT-3′) or the *UHRF1* distal promoter region (nucleotides −3217 to −3123; sense, 5′-CTCCCAAAGTGCTGGGATTA-3′, and antisense, 5′-GGCAACAAGAGCAAAACTCC-3′) were used. The primer concentration used for real-time PCR was 0.2 μM/25 μl. The thermal cycler conditions were as follows: 15 min of holding at 95°C followed by 45 cycles at 94°C for 15 s, 60°C for 30 s and 72°C for 30 s (Bio-Rad).

### Bisulfite DNA sequencing analysis

Genomic DNA (500 ng) was treated with sodium bisulfite using an EZ DNA Methylation kit (Zymo Research) following the manufacturer's protocol. The CpG sites of UHRF1, including the top genomic sequence in the DNA methylation array, was amplified from the bisulfite-treated genomic DNA using the following primers: BS-UHRF1-F, 5′-GAATTCGATATTATGTGGATTTAG-3′; and BS-UHRF1-R, 5′-GGATCCGCGAAAAAAAAAATAACC-3′. PCR products were cloned into an RBC T&A cloning vector (RBC).

### Microarray analysis

For G9a target gene profiling, we used the Illumina HumanHT-12 v4 Expression BeadChip (Illumina), which includes a pool of unique bead types that correspond to 47,228 transcripts. Total RNA (0.55 μg) isolated from K562 cells stably expressing shCTL RNA and shG9a RNA were reverse-transcribed and amplified, according to the protocols described in the Illumina TotalPrep RNA Amplification Kit (Ambion). *In vitro* transcription was then carried out to generate cRNA (0.75 μg), which was hybridized onto each array and labeled with SA-Cy3 (FluoroLink™ Cy™3). The array was then scanned using the Illumina Bead Array Reader Confocal Scanner. Array data export processing and analysis were performed using Illumina GenomeStudio v2009.2 (Gene Expression Module v1.5.4). This data set was submitted to the Gene Expression Omnibus under submission number GSE61610. For real-time PCR, total RNA (2 μg) was used to synthesize cDNA. cDNA synthesis was primed using an oligo (dT) primer (Fermentas), and the quantified cDNA was used for *G9a* and *UHRF1* mRNA expression pattern analysis. The primer sequences were as follows: for *hG9a*, 5′-ACGAGTGCAACTCCCGCTGC-3′ (sense) and 5′-GGCAGCCGCTCGTCAAGGTT-3′ (antisense); for *hUHRF1*, 5′-GTCGGATCATCTTCGTGGAC-3′ (sense) and 5′-AGTACCACCTCGCTGGCAT-3′ (antisense); for *mG9a*, 5′-GAGTGTAACCAGGCATGCTC-3′ (sense) and 5′-GCAGATGAACGTGCCCTG-3′ (antisense); and for *mUHRF1*, 5′-CGTGAACTCTCTGTCCAG-3′ (sense) and 5′-GTCATTGAGGCGCACATC-3′ (antisense). The amplification reaction was performed under the following conditions: 45 cycles of denaturation at 94°C for 15 s, annealing at 60°C for 30 s and extension at 72°C for 30 s. Dissociation curves were generated after each PCR run to ensure that a single product of the appropriate length was amplified. The mean threshold cycle (*C_T_*) and standard error were calculated from individual *C_T_* values obtained from three replicates per stage. The normalized mean *C_T_* was estimated as Δ*C_T_* by subtracting the mean *C_T_* of β-actin from those of G9a and UHRF1. ΔΔ*C_T_* was calculated as the difference between the control Δ*C_T_* and the values obtained for each sample. The fold-change in gene expression, relative to the untreated control, was calculated as 2^−ΔΔ *CT*^.

### FACS analysis

To measure the cell-cycle profile, G9a^−/−^ MEF and 293T H3K23R mutant stable cells were trypsinized, washed and fixed in ice-cold 70% ethanol for 30 min. Immediately before flow cytometric analysis, the cells were treated with RNase (100 μg/ml) and stained with propidium iodide (PI, Sigma) for 30 min, then subjected to fluorescence-activated cell sorting (FACS) analysis using a BD Accuri C6 cytometer (BD Biosciences). Data were analyzed using BD Accuri C6 software (BD Biosciences). To further measure the effect of G9a on the cell-cycle profile, stable G9a knockdown H1299 cells were transfected with GFP-G9a plasmids using Lipofectamine 2000 (Invitrogen) and were harvested 48 h after transfection.

### Antibodies

Antibodies specific for UHRF1 (sc-373750), Pol II (sc-899X), β-actin (sc-47778), LMO2 (sc-10497), Ub (sc-166553), HDAC1 (sc-7872), histone H3 (sc-8654), YY1 (sc-7341; Santa Cruz Biotechnology), G9a (07-551), H3K9-me2 (07-441), H3K27-me3 (07-449; Millipore), HDAC2 (ab12169), Acetyl-H3 (ab47915; Abcam), Flag (F3165; Sigma) and UHRF1 (GTX113963; GeneTex) were purchased.

### Immunohistochemistry and tissue array

Formalin-fixed paraffin-embedded tissue array slides containing various cancerous and normal tissues were purchased from Super Biochips. Briefly, after deparaffinization in xylene and rehydration in grade ethanol, endogenous peroxidase activity was blocked by incubation with 3% hydrogen peroxide for 10 min. Tissue sections were next heated in 100 mM citrate buffer (pH 6.0) for 10 min to retrieve antigens and then preincubated with normal horse serum for 20 min at room temperature. Anti-UHRF1 and anti-G9a antibodies (diluted 1:100) were used as the primary antibodies, and the specimens were subsequently incubated with biotinylated anti-rabbit secondary antibody (Vectastain Laboratory) and streptavidin-horseradish peroxidase (Zymed Laboratories Inc.). 3,3-Diaminobenzidine (DAB; Vectastain Laboratory) was used as a chromogen, and Meyer's hematoxylin was used for counterstaining. To have a negative control, the same procedure was followed with additional tissues, except that the primary antibody was replaced by PBS. The levels of G9a and UHRF1 expression were calculated by staining intensity using Quantity One software (Bio-Rad).

### DNA methylation profiling

Mononuclear cells were obtained from the bone marrow of eight leukemic childhood patient samples taken at the time of diagnosis. Three patients had precursor B cell-type acute lymphoblastic leukemia (ALL), one patient had T cell-type ALL, one patient had mixed phenotype (T/myeloid) leukemia, one patient had early precursor B cell-type ALL, and two patients had acute myeloid leukemia (AML). Informed consent was obtained from the guardians of each patient, and study approval was obtained from the Institutional Review Board of Chonnam National University Hospital. Genomic DNA was isolated from the childhood leukemia patient samples and normal samples. In all, 500 ng of input gDNA was bisulfite-converted according to the protocols in the Zymo EZ DNA methylation kit, which leave methylated cytosine unchanged but convert unmethylated cytosine to uracil. A total of 200 ng of input bisulfite-converted DNA was processed on an Illumina Infinium Human Methylation 27 BeadChip array (Illumina) to analyze the methylation status of 27,578 CpG sites. The whole-genome amplified DNA was fragmented, precipitated, resuspended, and incubated for a minimum of 16 h at 48°C, which allowed CpG loci to hybridize. Following hybridization, the allele-specific single-base extension assay was performed, and the slides were imaged on an Illumina BeadArray Reader, which is a two-color (543 nm/643 nm) confocal fluorescent scanner with 0.84 μm pixel resolution. The image intensities were extracted using Illumina's BeadScan software. This data set was submitted to the Gene Expression Omnibus under submission number GSE61611.

### Raw data preparation and statistical analysis

Raw data were extracted as β-values for each CpG in each sample using the software provided by the manufacturer (Illumina GenomeStudio v2010 (Methylation Module)). β-values were calculated by subtracting background using negative controls on the array and taking the ratio of the methylated signal intensity against the sum of both the methylated and unmethylated signals. A β-value of 0–1.0 was reported as the significant percentage of methylation, from 0 to 100%, respectively, for each CpG site (32). Array CpG probes with detection *P*-values ≥0.05 (similar to signal to noise) in more than 25% of samples were filtered out. Filtered data were normalized by the quantile method to reduce systemic bias. Statistical significance of the methylation data was determined using independent t-tests in which the null hypothesis was that no difference exists between the two clinical groups. The false discovery rate (FDR) was controlled by adjusting the *P*-value using the Benjamini–Hochberg algorithm. For differentially methylated CpGs, hierarchical cluster analysis was performed using complete linkage and Euclidean distance as a measure of similarity. Gene-enrichment and functional annotation analysis for the significant CpG site list was performed using PANTHER (http://www.pantherdb.org/panther/ontologies.jsp). All data analysis and visualization of differentially methylated CpGs was conducted using R 2.10.0 (www.r-project.org).

### Oncomine database analysis

The use of the database was described previously (33). Gene expression data for UHRF1 were retrieved from the Oncomine website (www.oncomine.org). Data sets with differential expression of UHRF1 between normal and cancerous tissues were selected.

### Statistical analysis

Data are expressed as means ± SDs of three or more independent experiments. Statistical significance (*P* < 0.05) was calculated using functions in Microsoft Excel. Differences between groups were evaluated by one-way analysis of variance (ANOVA), followed by a Student's *t*-test or Bonferroni test, as appropriate.

## RESULTS

### G9a negatively regulates transcription of UHRF1

**Figure 1.**
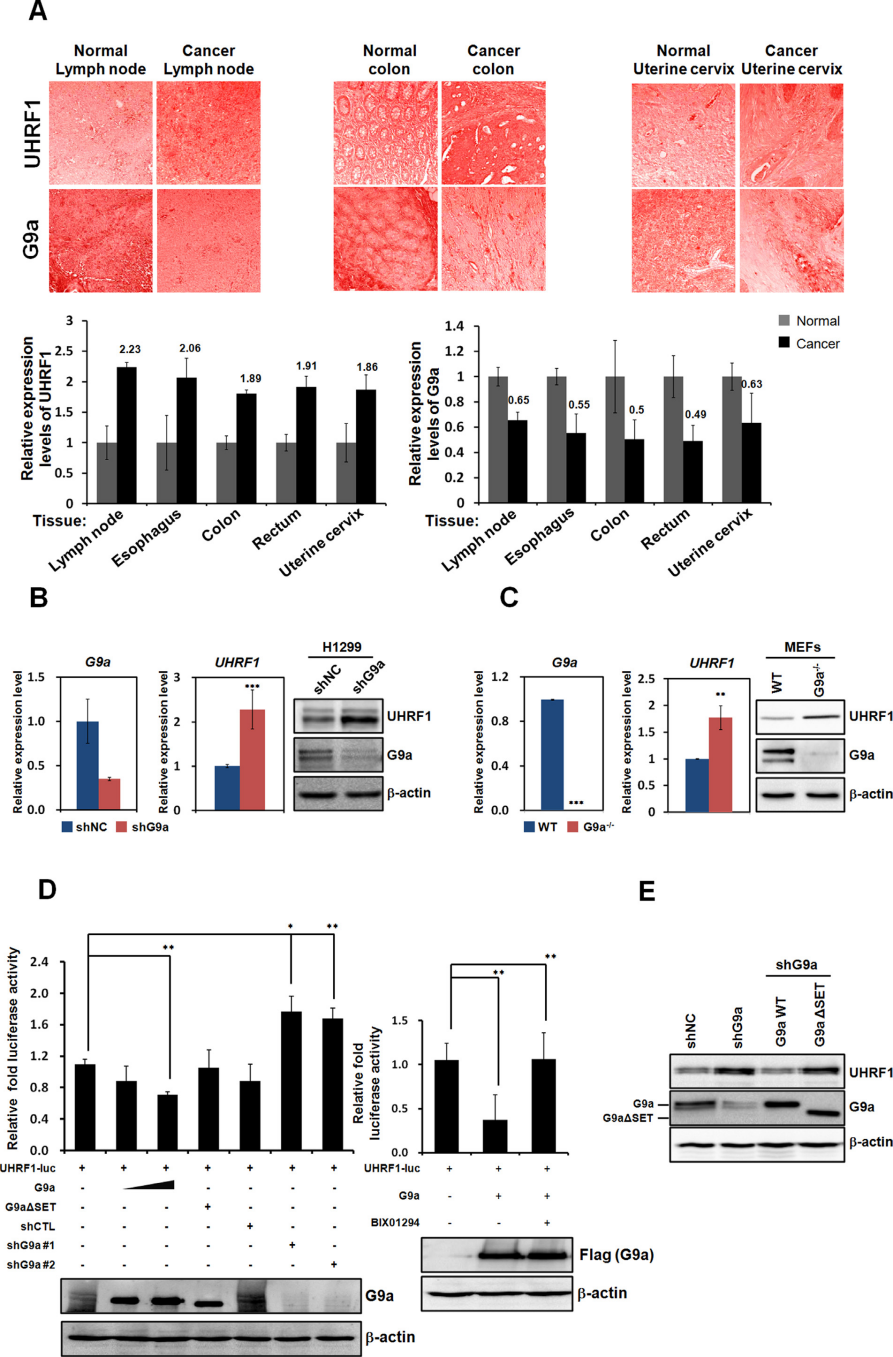

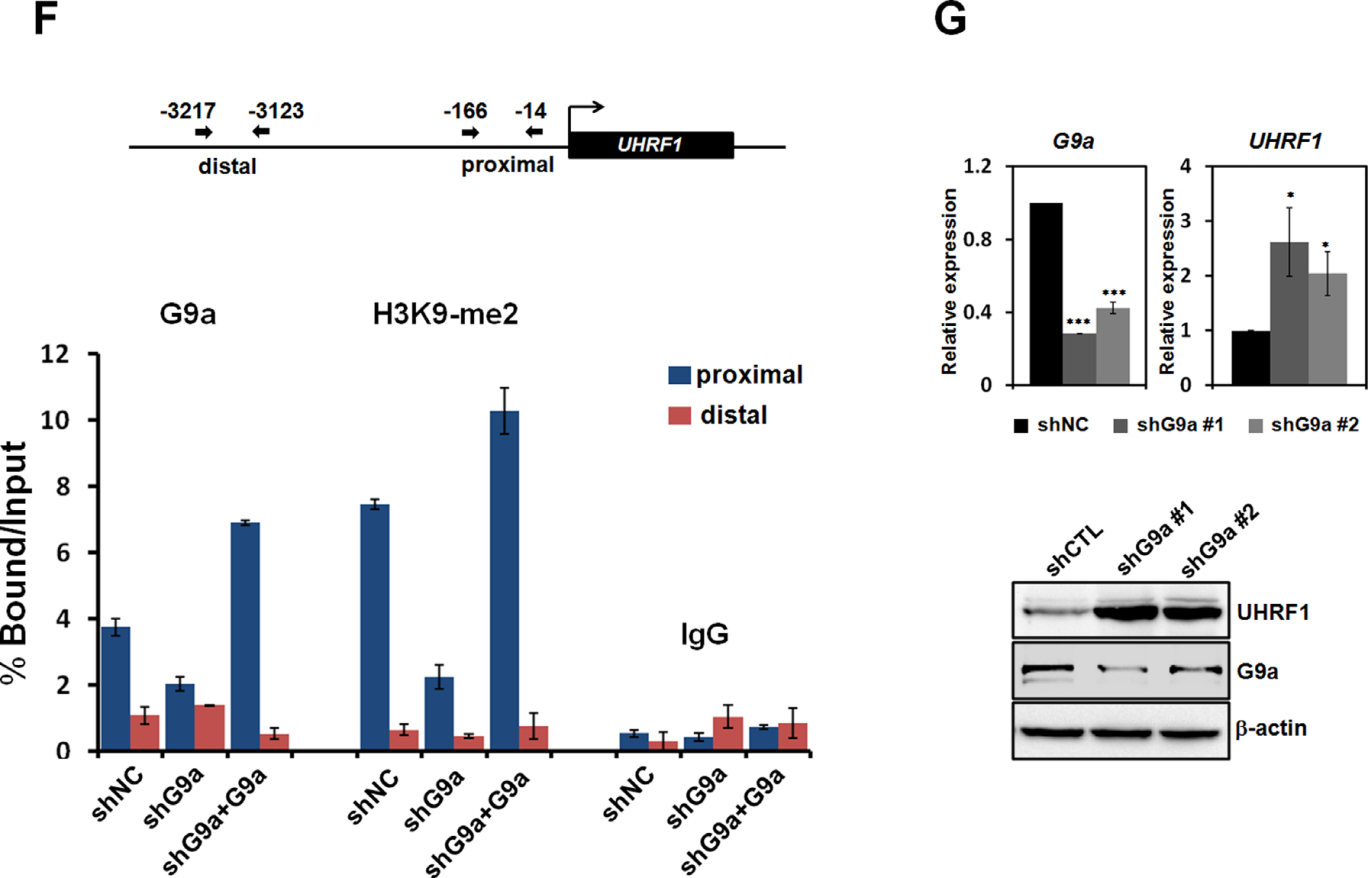
G9a negatively regulates UHRF1 transcription. (**A**) Formalin-fixed tissue array slides were used in immunohistochemistry experiments. Tissue array slides from lymph nodes, esophagus, colon, rectum and uterine cervix were used for immunohistochemical 3,3-diaminobenzidine (DAB) staining for G9a or UHRF1. Numbers in the graphs represent fold-changes relative to normal tissues. (**B** and **C**) G9a and UHRF1 mRNA levels were analyzed using real-time PCR in stable G9a-knockdown H1299 cells and G9a^−/−^ MEF cells. Cells were lysed and immunoblotted with anti-G9a and anti-UHRF1 antibodies. β-actin was used as a loading control. Results are shown as means ± SDs; *n* = 3. **P < 0.01, ***P < 0.001. (**D**) 293T cells were transfected with the pGL3-*UHRF1* promoter reporter and the indicated DNA constructs and shRNAs, and their cell extracts were assayed for luciferase activity (left panel). G9a-mediated UHRF1 restored transcriptional repression by BIX01294. The pGL3-*UHRF1* promoter and Flag-G9a were cotransfected into the 293T cells. Twenty-four hours after transfection, they were treated with BIX01294 (10 μM) for 24 h, and the luciferase activity was subsequently measured (right panel). Luciferase activity was normalized to that of β-galactosidase. Each *P*-value represents the mean of five replicates from a single assay. All results represent at least three independent experiments (±SD). *P < 0.05, **P < 0.01. Immunoblot analyses of the expression levels of endogenous and exogenous G9a in shG9a construct 1- or 2-transfected 293T cells are shown using anti-G9a, anti-Flag, and anti-β-actin antibodies. (**E**) Immunoblot analyses show the relative expression levels of UHRF1 in G9a knockdown and G9a WT and ΔSET rescued H1299 cells. The expression levels are normalized to β-actin. (**F**) Schematic diagram of primer pairs in ChIP analysis (upper panel). Arrows indicate the primers used for real-time PCR amplification. ChIP analyses of the *UHRF1* promoter in stable G9a knockdown H1299 cells were conducted using anti-G9a and anti-H3K9-me2, which were examined via real-time PCR. Results are shown as means ± SDs; *n* = 3. (**G**) The expression levels of G9a and UHRF1 in K562 cells with transient G9a knockdown were detected via real-time PCR and immunoblot analysis. The error bars represent 2^−ΔΔ*CT*^ ± the SD of three independent experiments. *P < 0.05, ***P < 0.001.

Previous studies suggest that UHRF1 is overexpressed in different types of cancers (26–29). Our search of the Oncomine database found that UHRF1 was upregulated in various types of cancers compared with its expression in normal tissues (Supplementary Figure S1A). To further delineate the expression levels of UHRF1 in different cancer tissues, we performed immunohistochemical analysis of a tissue array that contained both normal and cancerous tissues. UHRF1 expression was higher in certain types of the cancer tissues (12 tissues out of 16 tissues examined) than in normal tissues (Figure 1A). Among cancer tissues that highly express UHRF1 (compared with normal tissues), we found lymph node, colon, and cervical cancers by immunohistochemical staining (Figure 1A upper panels). Despite increasing findings about the oncogenic properties of UHRF1, little is known about its transcriptional regulation. Among different epigenetic regulators of UHRF1 transcription, we focused on HMTases related to transcriptional repression. Among different HMTases which have a role in the transcriptional repression of target genes, we first examined the expression levels of G9a by tissue array. Interestingly, among the tissues in which we observed UHRF1 upregulation, lymph node, colon and cervical cancers exhibited G9a downregulation (Figure 1A). Therefore, we hypothesized that upregulated UHRF1 expression might be correlated with G9a expression in different types of cancers, including leukemia.

To investigate the epigenetic transcriptional regulation of UHRF1, we first tested whether H3K9 histone methyltransferase G9a affects UHRF1 transcription. Real-time PCR and western blot analysis confirmed that knockdown of G9a (using the H1299 shG9a stable cell line) activated UHRF1 expression more than two-fold (Figure 1B). Regulation of UHRF1 expression by G9a was also demonstrated in G9a-deficient mouse embryonic fibroblasts (MEFs) using real-time PCR and western blot analysis (Figure 1C). Consistent results were produced in the stable shG9a K562 cell line (Supplementary Figure S1B). Next, we conducted a reporter assay using a *UHRF1*-luc reporter system to examine G9a-mediated transcriptional regulation of UHRF1. Consistent with the real-time PCR and western blot results, UHRF1 transcription was repressed by G9a overexpression in 293T cells (Figure 1D). Using the SET domain-deleted G9a mutant, we verified that transcriptional repression of UHRF1 by G9a was dependent on HMTase activity. G9a knockdown by two independent G9a shRNAs eliminated transcriptional repression of UHRF1 mediated by G9a (Figure 1D). These findings indicate that G9a negatively regulates UHRF1 transcription by directly acting on the *UHRF1* promoter. To confirm the role of G9a in negative regulation of UHRF1 transcription, we examined the effects of BIX01294, a G9a-specific inhibitor. That result further confirmed G9a-mediated transcriptional repression of UHRF1 (Figure 1D). Using an H1299 shG9a stable cell line, we confirmed that ectopically expressed G9a WT reduced UHRF1 expression. On the other hand, SET domain-deleted G9aΔSET failed to repress UHRF1 expression (Figure 1E). To further understand the mechanisms underlying UHRF1 transcriptional regulation via G9a, we performed chromatin immunoprecipitation (ChIP) analysis with real-time PCR using stable H1299 shG9a cells. We observed decreased G9a recruitment as well as decreased levels of H3K9-me2 on the *UHRF1* promoter when shG9a was introduced. On the other hand, G9a was highly recruited to the *UHRF1* promoter and H3K9-me2 levels increased when G9a was overexpressed, confirming the possibility of G9a recruitment to the *UHRF1* promoter (Figure 1F and Supplementary Figure S1F). Altogether, these results indicate that G9a negatively regulates the transcription of UHRF1 via recruitment to the promoter region. To further investigate the effects of histone H3K9 methyltransferase G9a on the transcription of target genes in leukemia cells, we performed global gene expression profiling with human erythroleukemia K562 cells stably expressing either control shRNA or G9a shRNA in a Tet-on inducible expression system (Supplementary Figure S1C). Two independent shG9a RNAs and control shRNA samples were analyzed using a microarray. A total of 185 genes were either up- or downregulated more than 1.5-fold when G9a was depleted (Supplementary Figure S1C). Notably among the genes regulated by G9a, *UHRF1* was found to be upregulated in the microarray analysis when G9a was depleted (Supplementary Figure S1C). In a biological function analysis using the PANTHER classification (http://www.pantherdb.org), these genes were shown to be associated with several major functional groups, including signal transduction, immunity and defense, nucleotide and nucleic acid metabolism, developmental processes, and the cell cycle (Supplementary Figure S1D). Furthermore, these differentially expressed genes were shown to be associated with other major groups, including nucleic acid binding, transcription factors and receptors using molecular functional analysis (Supplementary Figure S1D). Two different shG9a RNA samples used in the microarray were tested for their upregulation of UHRF1 (Supplementary Figure S1E). Furthermore, using erythroleukemia type K562, downregulation of UHRF1 transfected with two different shG9a RNAs was further confirmed by real-time PCR and western blot analysis (Figure 1G). Taken together, these results suggest that UHRF1 is a transcriptional target of G9a and that the activity of G9a results in the downregulation of UHRF1.

### YY1 functions as a corepressor in G9a-mediated downregulation of UHRF1 transcription

**Figure 2.**
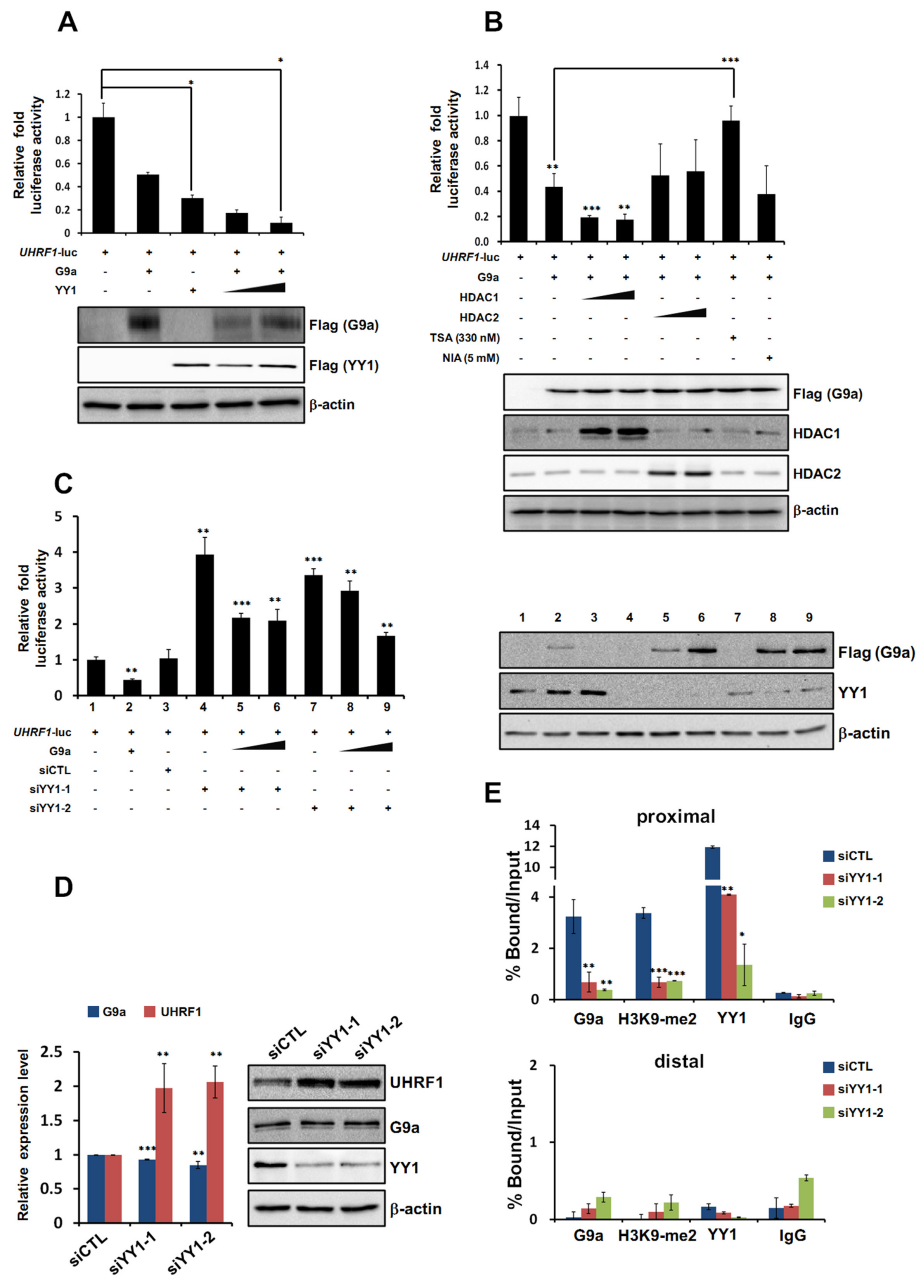
G9a-mediated transcriptional repression of UHRF1 is YY1 dependent. (**A**) 293T cells were cotransfected with the pGL3-*UHRF1* promoter, Flag-G9a and Flag-YY1. Following transfection, cells were grown for 48 h, and cell extracts were assayed for luciferase activity. Expression of the transfected constructs is shown in the immunoblot analysis. (**B**) pGL3-*UHRF1* promoter and the indicated constructs were cotransfected into 293T cells. Twenty-four hours after transfection, 330 nM TSA or 5 mM NIA was added for 24 h, and luciferase activities were subsequently measured. G9a, HDAC1 and HDAC2 expression was confirmed by immunoblot analysis. (**C**) 293T cells were cotransfected with the pGL3-*UHRF1* promoter, Flag-G9a, siCTL RNA and siYY1 RNAs (100 nM). Luciferase activity was measured 48 h after transfection. G9a overexpression and YY1 knockdown by two different siYY1 RNAs are shown in the immunoblot analysis. (**A**–**C**) Luciferase activity was normalized to that of β-galactosidase, and the results are presented as means ± SD (*n* = 3). *P < 0.05, **P < 0.01, ***P < 0.001. (**D**) 293T cells were transfected with siCTL RNA or siYY1 RNAs (100 nM). YY1, G9a, and UHRF1 expression levels were confirmed using real time-PCR and immunoblot analysis. YY1 knockdown by siYY1 RNA is shown in the immunoblot analysis. All results are representative of at least three independent experiments (±SDs). **P < 0.01, ***P < 0.001. (**E**) 293T cells were transfected with siCTL RNA or siYY1 RNAs. ChIP analysis was performed using anti-G9a, anti-YY1, and anti-H3K9-me2 antibodies, and the results were confirmed by real-time PCR. Recruitment of G9a, YY1 and H3K9-me2 to the *UHRF1* promoter and distal region was normalized by input. All results represent at least three independent experiments (±SDs). *P < 0.05, **P < 0.01, ***P < 0.001.

We further analyzed the *UHRF1* promoter sequence to identify possible transcription factor binding sites. Among them, we found that 5 YY1 binding sites (−12, −40, −840, −1097 and −1175 sites in the *UHRF1* promoter) were clustered between the −1921 and +145 sequences in the *UHRF1* promoter region, indicating that YY1 could be involved in UHRF1 transcription. As a ubiquitous and multifunctional Polycomb-group protein family transcription factor, YY1 plays critical roles in hematopoiesis and cell cycle control (34). We determined whether YY1 had a synergistic effect with G9a on the negative regulation of UHRF1 expression. As expected, the luciferase reporter assay showed that G9a and YY1 alone each repressed UHRF1 transcription. G9a cotransfection and an increase in YY1 further repressed UHRF1 transcription, suggesting that YY1 functions as a mediator for G9a recruitment to the *UHRF1* promoter and as a direct repressor of transcription (Figure 2A). Cotransfection of HDAC1 (but not HDAC2) with G9a further repressed G9a-mediated UHRF1 repression, indicating that HDAC1 plays a role in corepression (Figure 2B). Adding Trichostatin A (TSA) restored UHRF1 transcriptional repression by G9a, strongly suggesting that HDAC1 was involved in G9a-mediated UHRF1 transcriptional repression. However, adding nicotinamide (NIA) did not affect G9a-mediated UHRF1 transcriptional repression, indicating that the sirtuin class of histone deacetylases was not involved in this process (Figure 2B). The role of YY1 in G9a-mediated transcriptional repression of UHRF1 was further investigated by performing the UHRF1-luc reporter assay in the presence of siYY1 RNA. Interestingly, YY1 knockdown by two independent siYY1 RNAs abolished the UHRF1 transcriptional repression induced by G9a (Figure 2C). These data strongly suggest that negative regulation of UHRF1 transcription by G9a is dependent on the presence of YY1. We used real-time PCR and western analysis to determine whether UHRF1 expression was influenced by the presence or absence of YY1. Downregulation of UHRF1 was dependent on YY1, as shown by increased UHRF1 expression under two different YY1 knockdown conditions (Figure 2D). Next, we used ChIP and real-time PCR analysis to evaluate whether YY1 played a role in G9a recruitment to the *UHRF1* promoter. The data shown in Figure 2E indicate that G9a was highly recruited to the *UHRF1* promoter, and that YY1 knockdown by two independent siYY1 RNAs reduced G9a recruitment along with YY1. These findings consistently demonstrate that G9a can repress UHRF1 via binding of the YY1 transcription factor to the *UHRF1* promoter. Previously, we reported that G9a and YY1 interact to mediate transcriptional repression of JAK2 (35). The finding that the level of H3K9-me2 on the *UHRF1* promoter is downregulated in the absence of YY1 confirms that G9a-mediated UHRF1 transcriptional repression is YY1 dependent (Figure 2E). Taken together, these results suggest that G9a mediates transcriptional repression of UHRF1 in a YY1-dependent manner.

### UHRF1 is downregulated upon differentiation of the leukemia cell line

**Figure 3.**
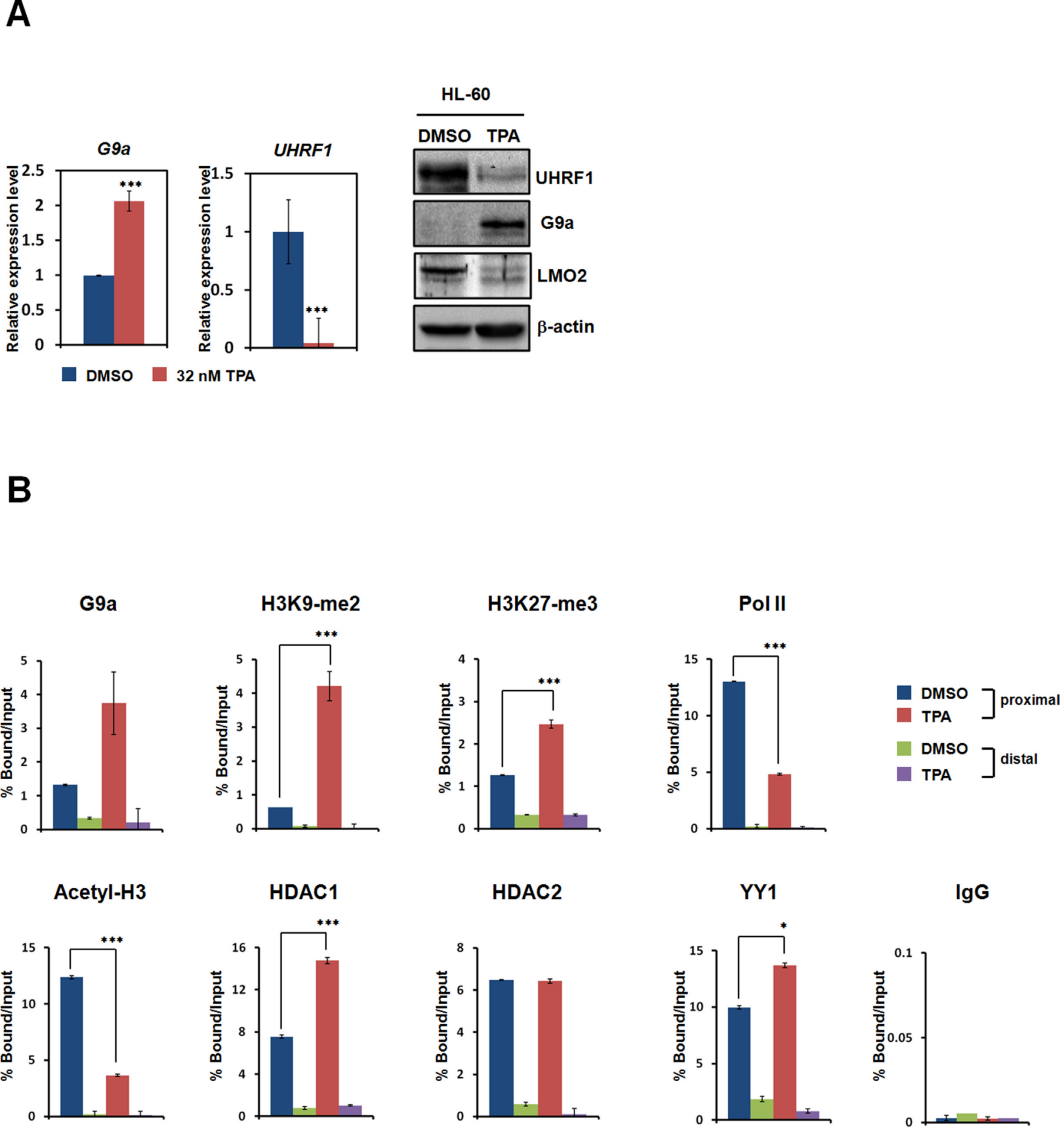
UHRF1 is downregulated by G9a during leukemia cell differentiation. **(A)** HL-60 cells were treated with TPA or DMSO. After 48 h, real-time PCR was performed to compare the expression levels of UHRF1. All results represent at least three independent experiments (±SDs). *** *P* < 0.001. Cells were lysed and immunoblotted with anti-G9a, anti-LMO2, and anti-UHRF1 antibodies. β-actin was used as a loading control. **(B)** ChIP analyses of the *UHRF1* promoter in TPA-treated HL-60 cells were conducted using anti-G9a, anti-H3K9-me2, anti-H3K27-me3, anti-Pol II, anti-Acetyl-H3, anti-HDAC1, anti-HDAC2, anti-YY1, and anti-IgG and were examined via real-time PCR analysis. All results represent at least three independent experiments (± SD). * *P* <0.05, *** *P* <0.001.

To investigate whether the level of UHRF1 expression changes during leukemia cell differentiation, we treated HL-60 with TPA and monitored UHRF1 expression patterns using real-time PCR and western analysis. Previously, we reported that G9a expression was upregulated after leukemia cell differentiation (35). Consistent with our hypothesis, UHRF1 expression was significantly downregulated after leukemic HL-60 cell line differentiation by TPA (Figure 3A). Additionally, expression of G9a was upregulated upon differentiation of the HL-60 cell line (Figure 3A). Leukemic oncogene protein LMO2 was also downregulated when HL-60 cells differentiated. This indicates transcriptional repression of UHRF1 upon leukemia cell differentiation. To examine the recruitment of G9a before and after leukemia cell differentiation, we performed ChIP assays and real-time PCR on the *UHRF1* promoter before and after TPA treatment. G9a recruitment to the *UHRF1* promoter increased after TPA treatment, and levels of H3K9-me2 and H3K27-me3 overlapped well with G9a, YY1, and HDAC1 recruitment (Figure 3B). Pol II and Acetyl-H3 were not recruited after TPA treatment, though their association with HDAC2 was unaffected (Figure 3B). Together, these results suggest that transcription of UHRF1 is downregulated by G9a during leukemia cell differentiation. Altogether, these ChIP and real-time PCR data support a transcriptional regulatory relationship between G9a and UHRF1.

### Hypomethylation of *UHRF1* promoter in leukemia patient samples

**Figure 4.**
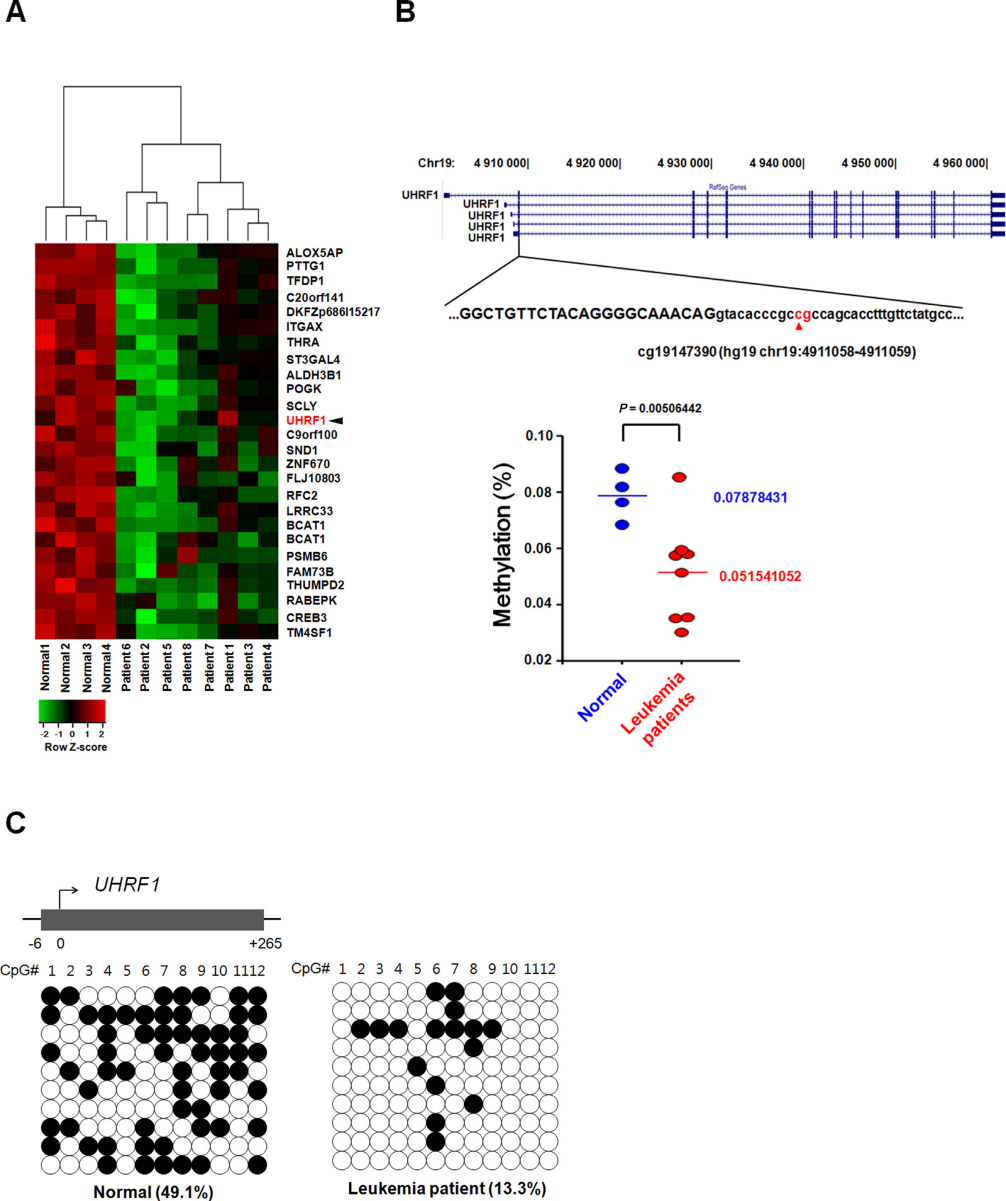
Hypomethylation of *UHRF1* promoter in leukemia patients. (**A**) A heat map of the differentially methylated CpG loci shows distinct patterns between data sets obtained from normal samples and those from leukemia patient samples. (**B**) Schematic diagram of the methylation probes used in the DNA methylation array (upper panel). Boxplots of DNA methylation levels on the *UHRF1* promoter show decreased levels in leukemia patient samples (lower panel). (**C**) Bisulfite DNA sequencing analysis was conducted to detect the DNA methylation status in the CpG sites of the *UHRF1* promoter in normal and leukemia patient samples. Closed circles indicate methylated CpGs, and open circles represent unmethylated CpGs. The percentage of DNA methylation (methylation CpG sites/total CpG sites) is given at the bottom of each panel.

Having identified G9a as an epigenetic transcriptional regulator of UHRF1, we next examined the DNA methylation status of UHRF1 in different leukemia patient samples. To compare the methylation status of gene promoters in leukemia patient samples and normal samples, we set up a DNA methylation array using blood samples from 8 leukemia patients. We used Infinium Methylation Assays (Illumina^®^ Human Methylation27 BeadChip) (36) with a platform containing 27,578 CpG sites covering 14,495 RefSeq genes, 12,833 annotated genes, 144 methylation hotspot genes, 982 cancer related genes, and 110 microRNA promoters. The analyzed CpGs were primarily located in the proximal promoter regions (and preferentially inside CpG islands, CGIs), and the array encompassed probes that covered an average of 1.9 CpG sites per promoter. To determine the biological relevance of the differential DNA methylation in leukemia patient samples, we carried out gene set enrichment analysis (Supplementary Figure S2) to identify functions epigenetically affected in leukemia. Next, we applied CGI prediction and analyzed whether a differential DNA methylation profile is located within or outside a CGI, which are widespread in distinct promoter classes. We found that about half of the differentially methylated CpG sites in leukemia patients (compared to those in normal samples) are located more than 2 kb from the nearest CGI (Supplementary Figure S2). Moreover, around half of the CpGs are located in CGI shores, which are defined as 1–2000 bp from a CGI border (37). Differential methylation profiles of promoters, promoter CGIs, and CGIs are shown in Supplementary Figure S2. Together, the analysis found a predominance of DNA methylation changes more than 2 kb away from CGIs, thus distinguishing the localization of the DNA methylation profile in leukemia patient samples from that in normal samples. We identified methylation changes by filtering the data sets for CpG sites that showed significant differences in DNA methylation levels between the normal and leukemia patient samples. The results of the filtering process are shown as a heat map (Figure 4A). By analyzing global DNA methylation profile between the normal and leukemia patient samples, we identified 446 gene promoters are hypermethylated and 411 gene promoters are hypomethylated in leukemia patient samples. Notably, among the genes distinguished between the two groups, the methylation status of the *UHRF1* promoter was downregulated in leukemia patient samples. The *UHRF1* promoter methylation status was measured using a methylation probe in an Infinium array (Figure 4B, upper panel). The boxplots of UHRF1 methylation status were decreased in leukemia patient samples compared to those in normal samples (Figure 4B, lower panel). Interestingly, UHRF1 was hypomethylated in ALL-type leukemia patient samples compared with AML-type leukemia patient samples. To confirm the methylation status of *UHRF1* CpG sites in leukemia patient samples through DNA methylation array, we produced a bisulfite sequencing primer for the same region of the DNA methylation array (Figure 4C, upper panel). Figure 4C depicts an example gene, *UHRF1*, for which the DNA methylation array data were confirmed by bisulfite sequencing. These results show that methylation status was lower in leukemia patient samples (13.3%) than in normal samples (49.1%). Consequently, our data are consistent with previous results that showed hypomethylation of the *UHRF1* promoter that resulted in higher expression in leukemia patient samples.

### G9a regulates UHRF1-mediated H3K23 ubiquitination and DNA replication

**Figure 5.**
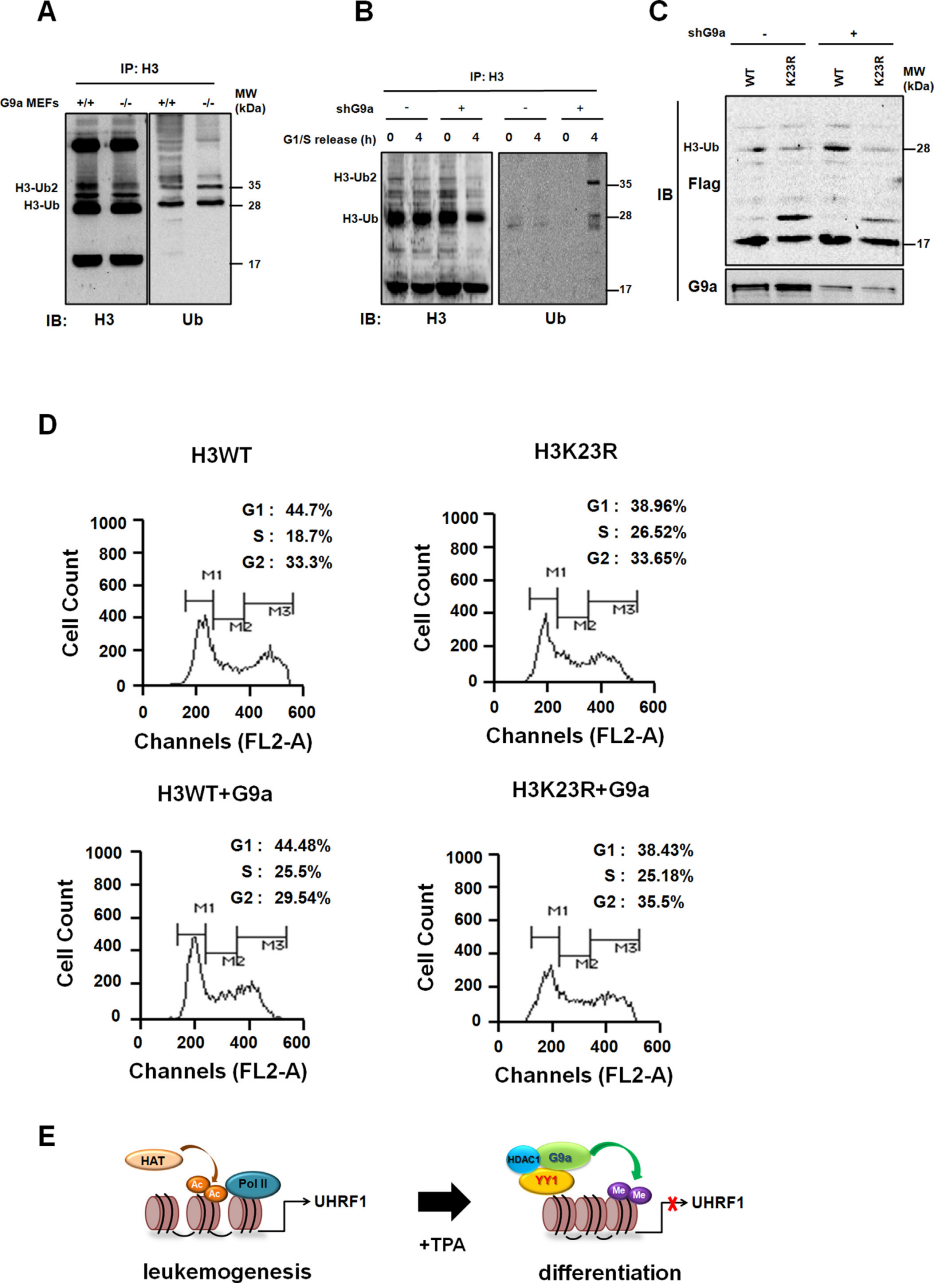
Regulation of UHRF1-mediated H3K23 ubiquitination and DNA replication maintenance. (**A**) Acid-extracted histones from asynchronous MEF cells were immunoprecipitated with anti-H3 antibody. The resultant immunoprecipitates were subjected to immunoblotting using anti-H3 and anti-Ub antibodies. (**B**) G9a knockdown H1299 cells were synchronized at G1/S and released into the S phase. Acid-extracted histones were subjected to immunoprecipitation using the anti-H3 antibody. Immunoprecipitates were analyzed by immunoblotting using anti-H3 and anti-Ub antibodies. (**C**) G9a knocked down in H1299 cells ectopically expressing either wild-type (WT) or K23R mutant Flag-tagged hH3. Cells were collected 48 h after transfection, and histone proteins were isolated by acid extraction. Cell extracts (bottom) and extracted histones (top) were subjected to immunoblotting using anti-G9a and anti-Flag antibodies, respectively. (**D**) Cell cycle progression in 293T H3 WT and H3K23R mutant stable cells was detected by PI staining. Cells were fixed, stained with PI for 30 min, and analyzed by FACS. (**E**) Model for regulation of UHRF1 transcription by G9a in leukemia cell differentiation.

UHRF1-dependent H3K23 ubiquitination is critical for the maintenance of DNA methylation (31). To further investigate the possible regulatory role of G9a on UHRF1-mediated H3K23 ubiquitination, we measured ubiquitinated H3 levels in G9a^−/−^ MEF cells. Western blot analysis clearly indicated elevated levels of ubiquitinated H3 in G9a^−/−^ MEF and stable G9a knockdown H1299 cells (Figure 5A and Supplementary Figure S3A). To verify that this increase in ubiquitinated H3 is dependent on the S phase, we synchronized G9a knockdown H1299 cells at the G1/S phase using thymidine and then released the cells into the S phase. As expected, ubiquitination of H3 was detected during the S phase, but not in the G1/S phase, which means G9a can regulate UHRF1-mediated H3 ubiquitination in the S phase (Figure 5B). Next, we examined whether lysine 23 is ubiquitinated in G9a knockdown cells. In those cells, we used anti-Flag antibodies in acid-extracted chromatin proteins and detected ectopically expressed H3 in cells expressing wild-type hH3, but not in cells expressing K23R mutant hH3, confirming that UHRF1-mediated ubiquitination has indeed occurred at the H3K23 site and is regulated by G9a (Figure 5C). Having established that G9a can modulate H3K23 ubiquitination by regulating UHRF1 expression, we next asked whether G9a can affect proper cell cycle progression, which is mediated by UHRF1-mediated H3K23 ubiquitination. Using PI staining, we conducted a FACS analysis in WT and G9a^−/−^ MEF cells and detected a decrease in S phase cells when G9a was knocked down (Supplementary Figure S3B). We obtained the same results with stable shNC and shG9a H1299 cell lines (Supplementary Figure S3C). The large decreases in the intensity of PI staining suggest that G9a knockdown led cells to faster S phase progression and impaired DNA replication. Next, we used a FACS analysis with H3 WT and H3K23R mutant stable cell lines to investigate the effect of G9a on UHRF1-mediated ubiquitination of H3K23 and the resulting effect on the regulation of cell cycle progression. More S phase cells were detected in the H3K23R stable cell line (26.52%) than in the H3 WT cell line (18.7%), which indicates that H3K23 ubiquitination is important for cell cycle progression, as previously reported (31) (Figure 5D). When G9a was ectopically expressed, the H3 WT cell line showed increased S phase cells (18.7 to 25.5%), suggesting delayed cell cycle progression in the S phase (Figure 5D). Expression of G9a didn't affect cell numbers in the S phase of the H3K23R mutation, which suggests the importance of H3K23 ubiquitination. Our data suggest that G9a regulates H3K23 ubiquitination via repression of UHRF1 expression, and that disruption impairs UHRF1-mediated ubiquitination and DNA replication during the S phase.

## DISCUSSION

G9a/GLP catalyze the mono- and dimethylation of the histone H3K9 and play key roles in regulating gene expression and chromosome structure during mammalian development (38). Recently, we reported a role for G9a as a negative regulator in JAK2 transcription during leukemia cell differentiation (35). In this paper, we further describe the extended role of H3K9 HMTase G9a in leukemia cells by showing its negative regulation of UHRF1 transcription. By identifying G9a as an upstream regulator of UHRF1 transcription, we propose its effect on UHRF1-mediated H3K23 ubiquitination and cell cycle progression. We used global gene expression profiling to identify UHRF1 as a target gene of G9a. The importance of the transcriptional regulation of UHRF1 during leukemia cell differentiation was further suggested by our ChIP and real-time PCR analysis. The decrease in Pol II and histone acetylation occupancies indicate that UHRF1 is transcriptionally repressed during leukemia cell differentiation. Moreover, hypomethylation of the *UHRF1* promoter in leukemia patients further suggests its higher expression in undifferentiated cellular conditions.

A recent genome-wide ChIP-seq analysis using G9a antibodies revealed that UHRF1 was strongly occupied by G9a in mouse embryonic stem cells (mESCs) (39) (Supplementary Figure S4A). It is interesting that genome-wide ChIP-seq analyses also identified no occupancy at the UHRF1 gene by Jarid2, Suz12, Ezh2 or H3K27-me3 in mouse ES cells, indicating a role for UHRF1 transcriptional activation during development (40). Another study demonstrated that Ezh2 was not recruited to the UHRF1 gene, but H2AK119 ubiquitination was strongly enriched at the UHRF1 transcription start site and the proximal region of the gene in a prostate cancer cell line, suggesting non-PRC2-mediated transcriptional repression of UHRF1 (41).

One study suggested that G9a was responsible for H3K56 monomethylation and hence maintenance of DNA replication via its association with PCNA (42). Interestingly, our data indicate that DNA replication was delayed in the presence of G9a overexpression. It is likely that both G9a-mediated H3K56 monomethylation and transcriptional regulation of UHRF1 play roles in DNA replication maintenance despite the different transcriptional strategies of early mESC and MEF cell lines.

Previous studies have linked UHRF1 with a number of cancers, including liver, prostate, colon, stomach and blood (26,28–30,43). To obtain further evidence of UHRF1 involvement in leukemogenesis, we searched the Oncomine database to compare the expression levels of UHRF1 in different types of leukemia with those in normal tissues. We found UHRF1 to be upregulated in leukemia compared to normal tissues (Supplementary Figure S4B). These data are consistent with the results from our experiments and strongly suggest that UHRF1 plays a role in leukemia and other carcinomas, possibly through G9a-mediated transcriptional regulation. We suggest that transcription of the oncogenic aspects of UHRF1 is activated by various leukemia-inducing conditions. On the other hand, TPA treatment induces leukemia cell differentiation by recruiting YY1 and G9a and represses transcription of UHRF1 via H3K9, a methylation-mediated silencing mechanism (Figure 5E). G9a has also been shown to repress the proto-oncogene TrkC/NTRK3, a receptor tyrosine kinase, and inhibit cellular transformation (44). Another study suggested that G9a interacts with Snail and transcriptionally represses metastasis-linked E-cadherin in human breast cancer cells (45). Moreover, G9a-mediated repressive epigenetic marks on H3K9-me2 were selectively enriched on the entire PPARγ gene locus during adipogenesis (46). A recent report suggests an oncogenic function for PPARγ in human thyroid carcinoma with G9a as a negative regulator of its oncogenic activity (47).

In summary, this study demonstrates that negative regulation of UHRF1 transcription by G9a is important in regulating the oncogenic activity of UHRF1 and leukemia cell differentiation. We propose that G9a has an upstream regulatory role in UHRF1-mediated H3K23 ubiquitination, maintenance of DNA replication through its epigenetic silencing machinery.

## SUPPLEMENTARY DATA

Supplementary Data are available at NAR Online.

SUPPLEMENTARY DATA
