# Supplementary material for: H3K9 methyltransferase G9a negatively regulates UHRF1 transcription during leukemia cell differentiation
Source: Nucleic Acids Res. 2015 Mar 12;43(7):3509–23. doi: 10.1093/nar/gkv183 (PMC4402520; doi:10.1093/nar/gkv183)
Supplement: SUPPLEMENTARY DATA [file supp_43_7_3509__index.html]

H3K9 methyltransferase G9a negatively regulates UHRF1 transcription during leukemia cell differentiation — SUPPLEMENTARY DATA 

# H3K9 methyltransferase G9a negatively regulates UHRF1 transcription during leukemia cell differentiation

## SUPPLEMENTARY DATA

**Files in this Data Supplement:**

- SUPPLEMENTARY DATA
