# Supplementary material for: H3K9 methyltransferase G9a negatively regulates UHRF1 transcription during leukemia cell differentiation
Source: Nucleic Acids Res. 2015 Mar 12;43(7):3509–23. doi: 10.1093/nar/gkv183 (PMC4402520; doi:10.1093/nar/gkv183)
Supplement: SUPPLEMENTARY DATA [file supp_gkv183_2015-02-02_Revision_Kim_et_al_Supplementary_Data-NAR.pdf]

SUPPLEMENTARY INFORMATION

H3K9 Methyltransferase G9a Negatively Regulates UHRF1 Transcription During Leukemia Cell Differentiation

Kee-Beom Kim[1], Hye-Ju Son[1], Sulji Choi[1], Ja Young Hahm[1], Hyeonsoo Jung[1], Hee Jo Baek[2], Hoon Kook[2], Yoonsoo Hahn[1], Hyun Kook[3] and Sang-Beom Seo[1,*]

[1]Department of Life Science, College of Natural Sciences, Chung-Ang University, Seoul 156-756, [2]Environmental Health Center for Childhood Leukemia and Cancer, Department of Pediatrics, Chonnam National University Hwasun Hospital, Hwasun 519-809, [3]Medical Research Center for Gene Regulation and Department of Pharmacology, Chonnam National University, Gwangju 501-746, Republic of Korea.

*To whom correspondence should be addressed. Tel.: 82-2-820-5242; Fax: 82-2-822-3059; Email: sangbs@cau.ac.kr

Supplementary Figure S1

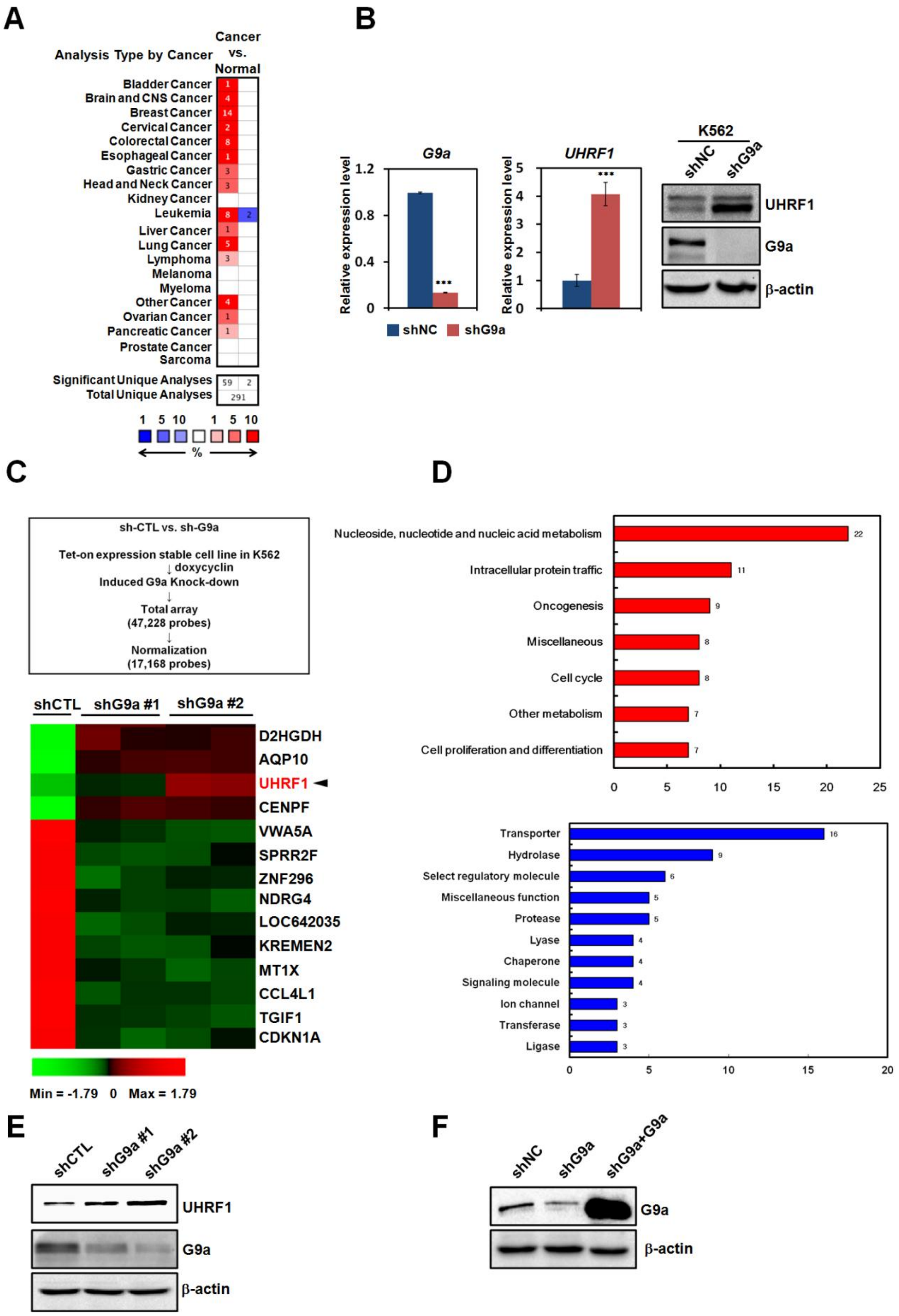

Supplementary Figure S2

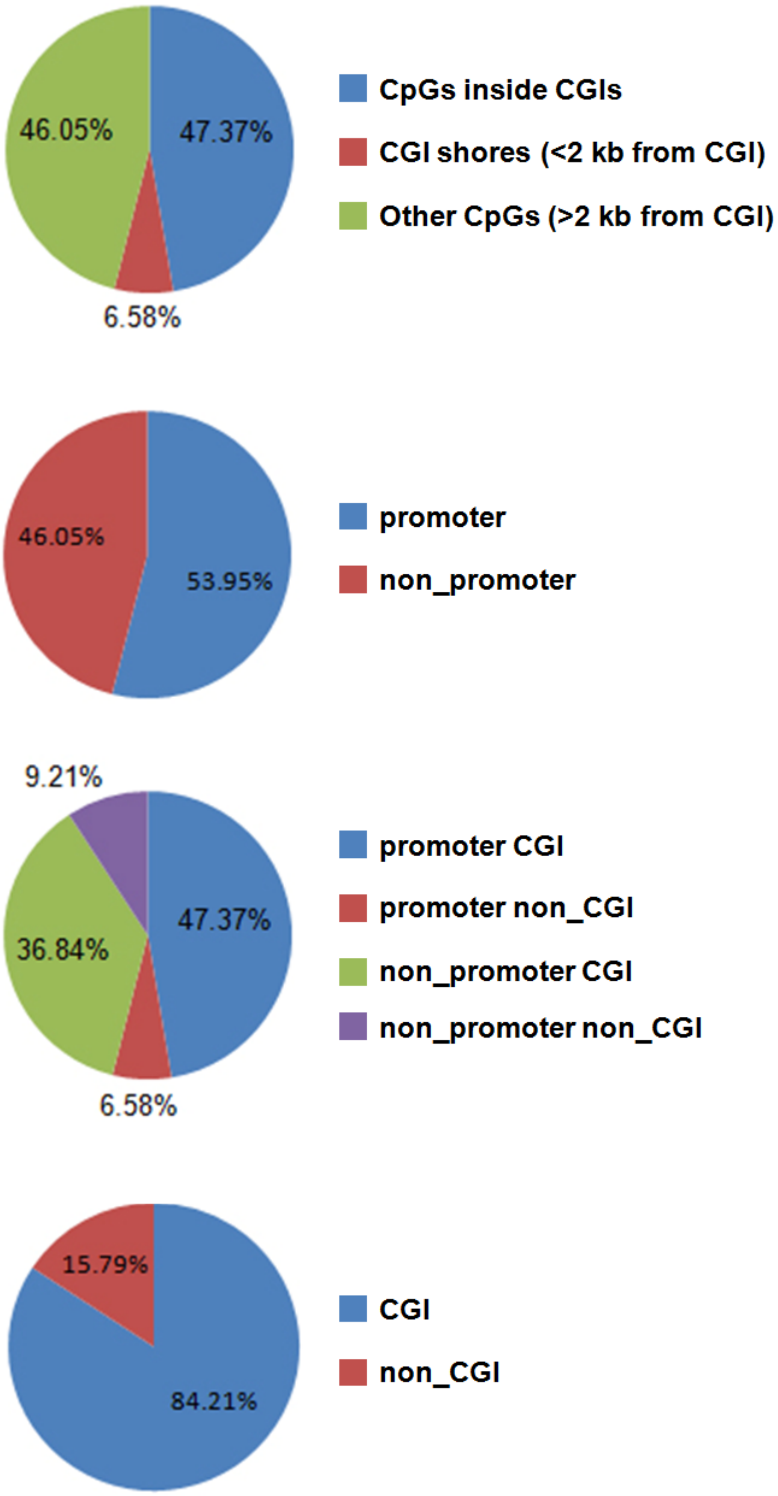

Supplementary Figure S3

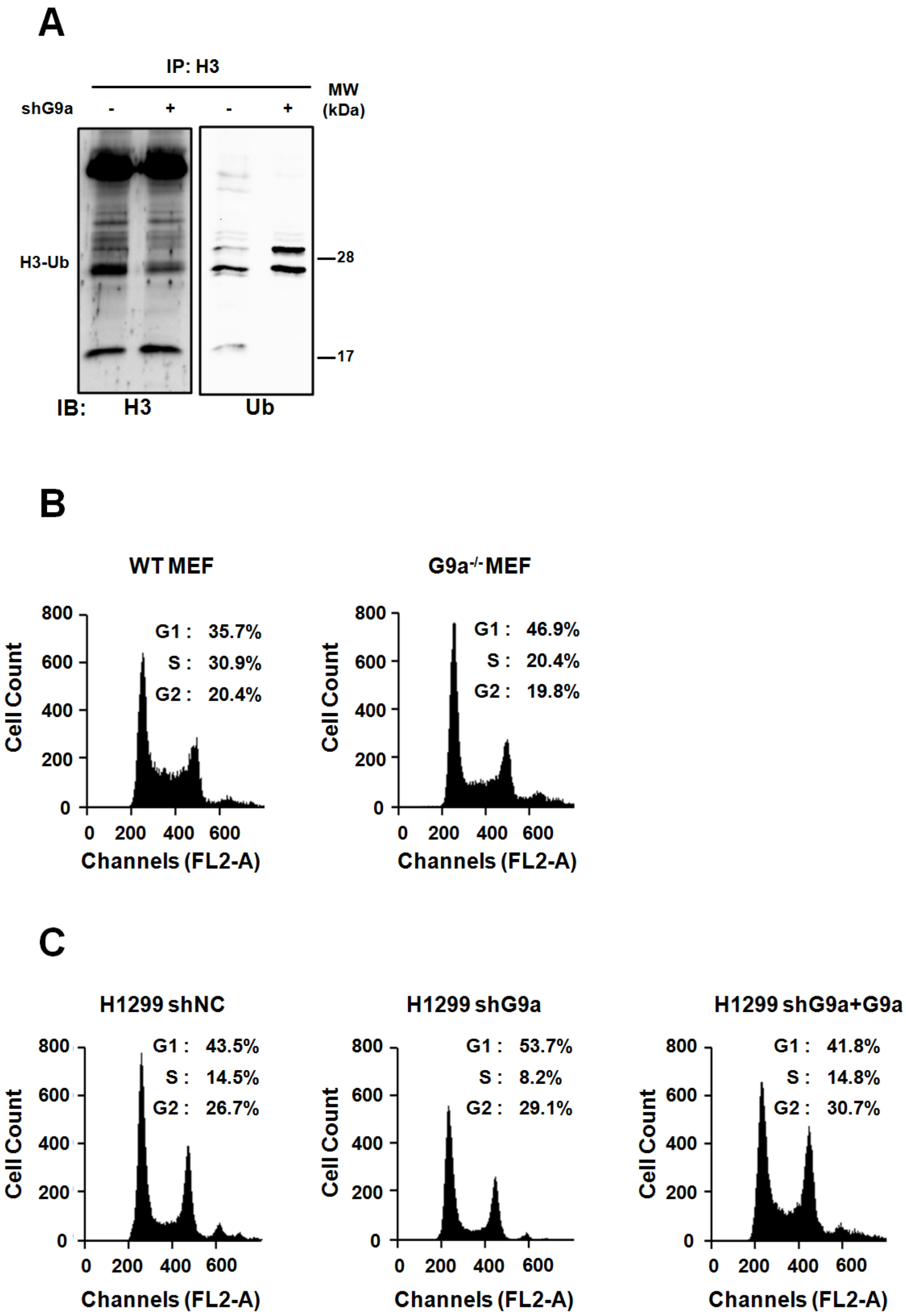

Supplementary Figure S4

A

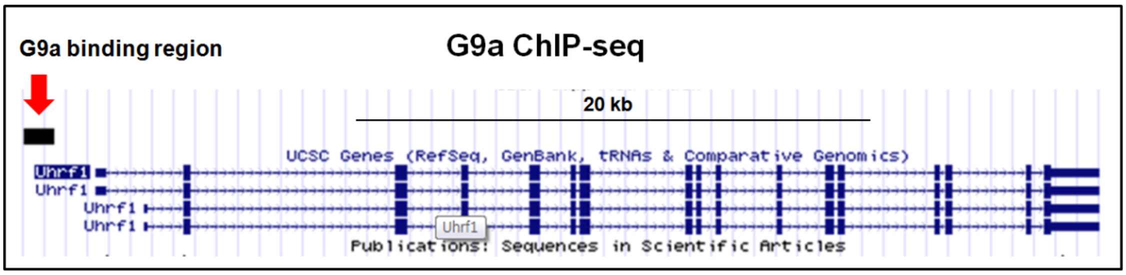

B

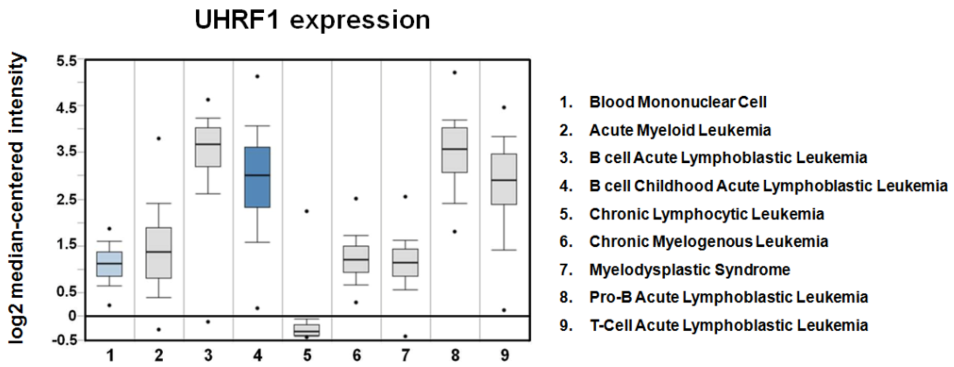

Supplementary Figure Legends

Supplementary Figure S1. G9a negatively regulates UHRF1 expression. **(A)** Oncomine analysis of UHRF1 expression in normal cells and various cancer. Red signifies the gene's over-expression, and blue represents the gene's under-expression in those analyses. The intensity of color signifies the best rank of that gene in those analyses, and the number in each cell represents the number of analyses that meet thresholds (*P*-value: 1E-4, Fold change: 2, Gene rank: Top 10%) within each analysis and cancer type. Cell color is determined by the best gene rank percentile for the analyses within the cell. **(B)** G9a and UHRF1 mRNA levels were analyzed using real-time PCR in stably G9a knockdown K562 cells. Cells were lysed and immunoblotted with anti-G9a and anti-UHRF1 antibodies. β-actin was used as the loading control. Results are shown as means ± SDs; n = 3. *** $P < 0.001$. **(C)** Flow chart showing the strategy used for the microarray analysis (upper panel) and G9a target gene identification. G9a target genes were identified by hierarchical clustering; expression changes in shCTL and stable shG9a knockdown K562 stable cells were compared. Upregulated and downregulated gene clusters are shown in red and green, respectively. **(D)** Biological and molecular functional classification of G9a target genes. (**E)** Stable shCTL or shG9a knockdown stable cells were lysed and immunoblotted with anti-G9a and anti-UHRF1 antibodies. β-actin was used as a loading control. **(F)** Immunoblot analyses showed the relative expression levels of UHRF1 in G9a knockdown and G9a rescued H1299 cells.

Supplementary Figure S2. DNA methylation profiling in leukemia patient samples. Classification of the promoter or CpG sites according to their location relative to CpG islands. Most of the differentially methylated CpG sites were affiliated with genes not possessing CpG or those > 2 kb away from the nearest CpG island (termed ‘other CpGs’). The affected

CpGs were located inside a CGI on CGI shores, that is, the distance to the CGI is between 1 and 2000 bp.

Supplementary Figure S3. Regulation of UHRF1-mediated H3K23 ubiquitination and DNA replication maintenance by G9a. **(A)** Acid-extracted histones from asynchronous stable G9a knockdown H1299 stable cells were immunoprecipitated with anti-H3 antibody. The resultant immunoprecipitates were subjected to immunoblotting using anti-H3 and anti-Ub antibodies. **(B)** and **(C)** Cell cycle progression in G9a$^{-/-}$ MEF cells and stable shG9a knockdown H1299 cells was detected by PI staining. Cells were fixed, stained with PI for 30 min, and analyzed by FACS.

Supplementary Figure S4. Genome-wide G9a distribution in G9a$^{-/-}$ mESC cells and Oncomine analysis of UHRF1 in leukemia. **(A)** An analysis of ChIP-seq data showing that G9a occupancy was enriched at the *UHRF1* promoter in mouse embryonic stem cells (1). **(B)** Boxplot of UHRF1 expression in normal and leukemia patient samples. *P*-values (4.16E-63) are calculated in comparison with column 1.

SUPPLEMENTARY REFERENCE

1. Mozzetta, C., Pontis, J., Fritsch, L., Robin, P., Portoso, M., Proux, C., Margueron, R. and Ait-Si-Ali, S. (2014) The histone H3 lysine 9 methyltransferases G9a and GLP regulate polycomb repressive complex 2-mediated gene silencing. *Mol Cell*, **53**, 277-289.
